# Effect of Three-Month Weight Training Program on Resting Heart Rate and Blood Pressure in Healthy Young Adult Males

**DOI:** 10.7759/cureus.34333

**Published:** 2023-01-29

**Authors:** Baidyanath Mishra, Vasanthan S, Himel Mondal

**Affiliations:** 1 Physiology, Sri Jagannath Medical College and Hospital, Puri, IND; 2 Physiology, Mahatma Gandhi Medical College and Research Institute, Puducherry, IND; 3 Physiology, All India Institute of Medical Sciences Deoghar, Rampur, IND

**Keywords:** hypertension, sympathetic, arterial stiffness, heart rate, isotonic exercise, weight training, young adults, blood pressure, endurance, exercise

## Abstract

Background

A planned and structured physical activity is the cornerstone of improving and sustaining body fitness. The underlying reason for exercise is personal interest, maintaining good health, or improving endurance for sports. Furthermore, exercise may be either isotonic or isometric. In weight training, different types of weight are being used and are lifted against gravity, and this type of exercise is of isotonic type.

Objective

The objective of this study was to observe the changes in heart rate (HR) and blood pressure (BP) after a three-month weight training intervention in healthy young adult males and to compare them with age-matched healthy control.

Materials and methods

We initially recruited a total of 25 healthy male volunteers for the study and 25 age-matched participants in the control group. Research participants were screened for any existing diseases and suitability for participation by the Physical Activity Readiness Questionnaire. We lost one participant from the study group and three participants from the control group in the follow-up. A structured weight training program (five days a week for three months) was applied for the study group with direct instruction and supervision in a controlled environment. A single expert clinician measured baseline and post-program (after three months) HR and BP (measured after 15 minutes, 30 minutes, and 24 hours of rest after exercise) to reduce any possible inter-observer variation. For comparing the pre-exercise and post-exercise parameters, we considered the post-exercise measurement, which was done after 24 hours of exercise. Mann-Whitney U test, Wilcoxon signed-rank test, and Friedman test compared the parameters.

Result

A total of 24 males with a median age of 19 years (Q1-Q3: 18-20) participated as the study group and 22 males with the same median age were the control group. At the end of the three-month weight training exercise program, there was no significant change in the HR (median 82 versus 81 bpm, p = 0.27) in the study group. The systolic BP was increased (median 116 versus 126 mmHg, p <0.0001) after three months of the weight training program. In addition, pulse pressure and mean arterial BP was also increased. However, diastolic (median 76 versus 80 mmHg, p = 0.11) BP was not significantly increased. There was no change in HR, systolic and diastolic BP in the control group.

Conclusion

A structured weight training program (used in this study) for three months may sustain an increase in systolic BP at rest in young adult males while diastolic BP remains the same. The HR remains unchanged before and after the exercise program. Hence, those enrolling in such an exercise program should be monitored frequently for changes in BP over time for any timely intervention appropriate for the candidate. However, being a small-scale study, the result of this study would be validated by further observing the underlying causes of the increment of systolic blood pressure.

## Introduction

Planned, structured, and regular physical activity is the cornerstone of improving and sustaining body fitness [1]. The World Health Organization has recommended certain sets of physical activity levels according to different age groups. For the adult population, the required physical activity is 150 to 300 minutes of moderate-intensity aerobic exercise or at least 75 to 150 minutes of vigorous-intensity exercise or equivalent energy expenditure per week. In addition to that, adults should engage themselves in muscle-strengthening exercises for two or more days a week [2]. However, all over the world, lack of space, education and employment systems, and changes in leisure activities have enforced many people to adopt a sedentary lifestyle [3].

Based on muscle length during exercise, the exercise may be divided into isometric exercise and isotonic exercise. In isometric exercise, the muscle length remains unchanged during the period of exercise, and the muscle length changes, but the tone remains unchanged during isotonic exercise [4]. In resistance training or weight training, different types of weight are being used and are lifted against gravity, and this type of exercise is of the isotonic type. The resistance exercise intervention may be used to increase strength and promote muscular growth. A healthy individual can also practice resistance training to reduce the amount of body fat and increase muscle mass. Because low body fat levels are linked to higher risks of several illnesses, including cardiovascular disease, resistance exercise not only helps promote muscle hypertrophy but also helps maintain better health [5].

A meta-analysis found that weight training significantly reduces BP among normotensive or pre-hypertensive subjects [6]. In contrast, other trials showed no significant changes in BP in hypertensive subjects [7]. Supporting this, Carter et al. found that a weight training program helped in reducing the systolic and diastolic BP in normotensive young adults [8]. In contrast, a meta-analysis found that weight training does not significantly change blood pressure in preadolescents and adolescents [9]. de Souza Nery et al. reported that systolic blood pressure is not increased in normotensive but is slightly elevated among hypertensive individuals during rest after weight training sessions [10].

Many young adult males prefer weight training for muscle hypertrophy. If their BP is raised due to the exercise program, this may be a health hazard. A 30-minute resting period is considered adequate for the measurement of blood pressure, according to the Centers for Disease Control and Prevention [11]. Hence, we aimed to measure the baseline HR, systolic, and diastolic BP and then start an intervention of weight training for three months period and then measure the parameters at 15 minutes, 30 minutes, and 24 hours after exercise to observe the changes. The study result would appraise us about the effect of short-term weight training exercise programs on resting heart rate and BP in young adult males.

## Materials and methods

Type and settings

This was an interventional study conducted in the Department of Physiology at Mahatma Gandhi Medical College and Research Institute located in the union territory of Puducherry. The study period was from June 2020 to May 2021.

Ethics

This study was approved by the Institutional Human Ethics Committee after a full board review by the Mahatma Gandhi Medical College and Research Institute, Sri Balaji Vidyapeeth, with approval number MGMCRI/RAC/02/2020/XX/IHEC/138. All the research participants were adults (age >18 years), and they provided written informed consent for voluntary participation. The participants were recruited with an advertisement for the exercise program. They were not provided any incentives; however, they were provided the exercise setting for free, including access to sports equipment and training by a qualified exercise trainer.

Participant recruitment

Young adult male (aged 18 to 25 years) volunteers were recruited for this study. The recruitment was done via an advertisement in the undergraduate medical college associated with the research centers. Only males were recruited due to the lack of female exercise trainers and the potentially low recruitment rate due to the psychosocial challenges of exercising females with males in a single setting. After the recruitment of the study group, the control was recruited with similar ages, heights, and weights. Any volunteer in the specified age range was initially enrolled and screened for participation suitability using the Physical Activity Readiness Questionnaire [12]. Subjects with any neuromuscular disease, suffering from hypertension and on medication, or with diabetes mellitus or any other metabolic diseases were excluded from the study.

Intervention

Weight training was provided to the study group for five days a week for three months under supervision. The exercise program consists of the chest, back, shoulder, hand, and leg muscle workouts, allotted on five different days a week. Each muscle group workout was done in five different forms using body weight, barbell, dumbbell, cable, and lever with three repetitions each, with increasing weights. Subjects were allowed two to three minutes of rest between every individual set of exercises. This program was selected for this study as it is used in this region to promote muscle growth.

Recording of heart rate and blood pressure

Pre-intervention measurement was done before exercise with a five-minute rest. Post-intervention measurement was done after the completion of the three-month program, after a rest of 15 minutes, 30 minutes, and 24 hours after exercise. The HR was measured manually by palpating the radial artery for three consecutive minutes by an expert clinician. The blood pressures were also measured manually by the same clinician three times with an aneroid sphygmomanometer and stethoscope. The blood pressure was measured in a sitting posture, with the left arm kept at the level of the heart. The back was supported, and the feet were flat on the floor. The highest value of three consecutive measurements with a gap of two minutes was taken as the final reading. The pulse pressure and mean arterial blood pressure were measured from the final reading. The weight and height were also measured by a digital weighing scale with 100 gm of sensitivity and a fiberglass measuring tape to the nearest 1 mm, respectively.

Statistical analysis

We used GraphPad Prism 6.01 for statistical analysis. A p-value of <0.05 was fixed as statistically significant. The data were first checked for normality by the Shapiro-Wilk test. As the data were not normally distributed, they were expressed in terms of the median and interquartile range (first quartile to third quartile). The Mann-Whitney U (non-parametric equivalent to an unpaired t-test) test was used to compare the variables between the study and the control group. The Wilcoxon signed-rank test (non-parametric equivalent to a paired t-test) was used for comparing pre-intervention and post-intervention (value that was obtained after 24 hours of rest) variables. The control group has no data after 15 minutes, 30 minutes, and 24 hours of rest; hence, only Wilcoxon signed rank was used. In contrast, the HR and BP before and after intervention at 15 minutes, 30 minutes, and 24 hours of rest were compared by the Friedman test (the non-parametric equivalent of a repeated measure ANOVA) with post hoc analysis. These non-parametric tests were considered as the data were not normally distributed.

## Results

**Figure 1:**
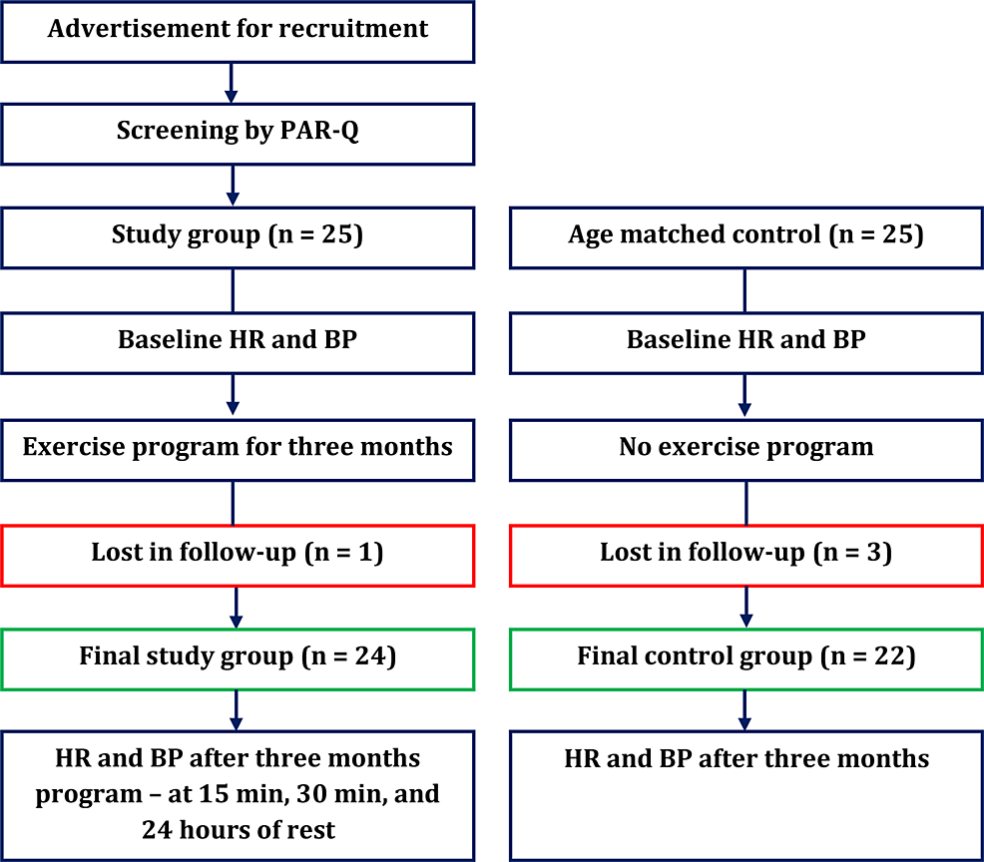
Study and control group research participants and brief study process PAR-Q: Physical activity readiness questionnaire, HR: Heart rate, BP: Blood pressure

A total of 24 young adult males with a median age of 19 years comprised the study group, and a total of 22 males with the same median age comprised the control group. One participant in the study group discontinued the exercise after one month, and three participants in the control group could not be included due to their unavailability. The study participants and study process are shown in Figure 1.

The age, height, weight, and BMI of the study group and control group participants are shown in Table 1. There was no significant difference in age and anthropometric parameters in the study and control groups.

**Table 1:** Age, height, weight, and body mass index of study and control group of research participants *p-value is of Mann-Whitney U test BMI: Body mass index

Parameter	Study group (n = 24)	Control group (n = 22)	p-value*
	Median (first quartile to third quartile)		
Age (years)	19 (18 to 20)	19 (18 to 20)	0.68
Height (cm)	174 (167.5 to 178.5)	172 (166 to 173.25)	0.23
Weight (kg)	67 (62 to 82)	65.5 (60.75 to 69.25)	0.15
BMI (kg/m^2^)	22.68 (21.29 to 24.98)	22.68 (19.8 to 24.67)	0.32

The HR, systolic BP, diastolic BP, pulse pressure, and mean arterial BP before the intervention are shown in Table 2. The heart rate among the study group was higher than the control group. Other parameters were similar in both groups.

**Table 2:** Measured variables in the study and control group of research participants before the intervention (exercise program) All the measurements were done with five minutes of rest before the exercise in the study group. Bpm: beats per minute, mmHg: millimeter of mercury *statistically significant p-value of Mann-Whitney U test

Variables	Study group (n = 24)	Control group (n = 22)	p-value
	Median (first quartile to third quartile)		
Heart rate (bpm)	82 (78 to 90)	74 (72.5 to 79)	0.0001*
Systolic blood pressure (mmHg)	116 (110 to 120)	116 (110 to 120)	0.7
Diastolic blood pressure (mmHg)	76 (72 to 80)	76 (70 to 80)	0.44
Pulse pressure (mmHg)	40 (32 to 44)	40 (36 to 46)	0.76
Mean arterial pressure (mmHg)	90 (84.67 to 94.33)	90 (83.17 to 93.5)	0.44

The HR, systolic BP, diastolic BP, pulse pressure, and mean arterial BP after the intervention are shown in Table 3. Post-intervention, too, the heart rate among the study group was higher than the control group. The systolic BP, diastolic BP, pulse pressure, and mean arterial BP were all raised in the study group.

**Table 3:** Measured variables in the study and control group of research participants after the intervention (exercise program) All variables in the study group were measured after 24 hours of rest. *Statistically significant p-value of Mann-Whitney U test. bpm: beats per minute, mmHg: millimeter of mercury

Variables	Study group (n = 24)	Control group (n = 22)	p-value
	Median (first quartile to third quartile)		
Heart rate (bpm)	81 (75.5 to 85)	72.39 (67.52 to 80.14)	0.003*
Systolic blood pressure (mmHg)	126 (118 to 130)	110 (100 to 122)	<0.0001*
Diastolic blood pressure (mmHg)	80 (72 to 82)	78 (66 to 80)	0.024*
Pulse pressure (mmHg)	46 (36 to 57)	40 (30 to 46)	0.016*
Mean arterial pressure (mmHg)	96.67 (89.33 to 99)	88.67 (78.67 to 93.67)	0.0003*

In control, the HR (p = 0.36), systolic BP (p = 0.28), diastolic BP (p = 0.91), pulse pressure (p = 0.52), and mean arterial blood pressure (p = 0.71) did not show any difference in the pre-intervention and post-intervention phases (measurement after 24 hours of rest) as statistically tested by the Wilcoxon signed rank test.

**Figure 2:**
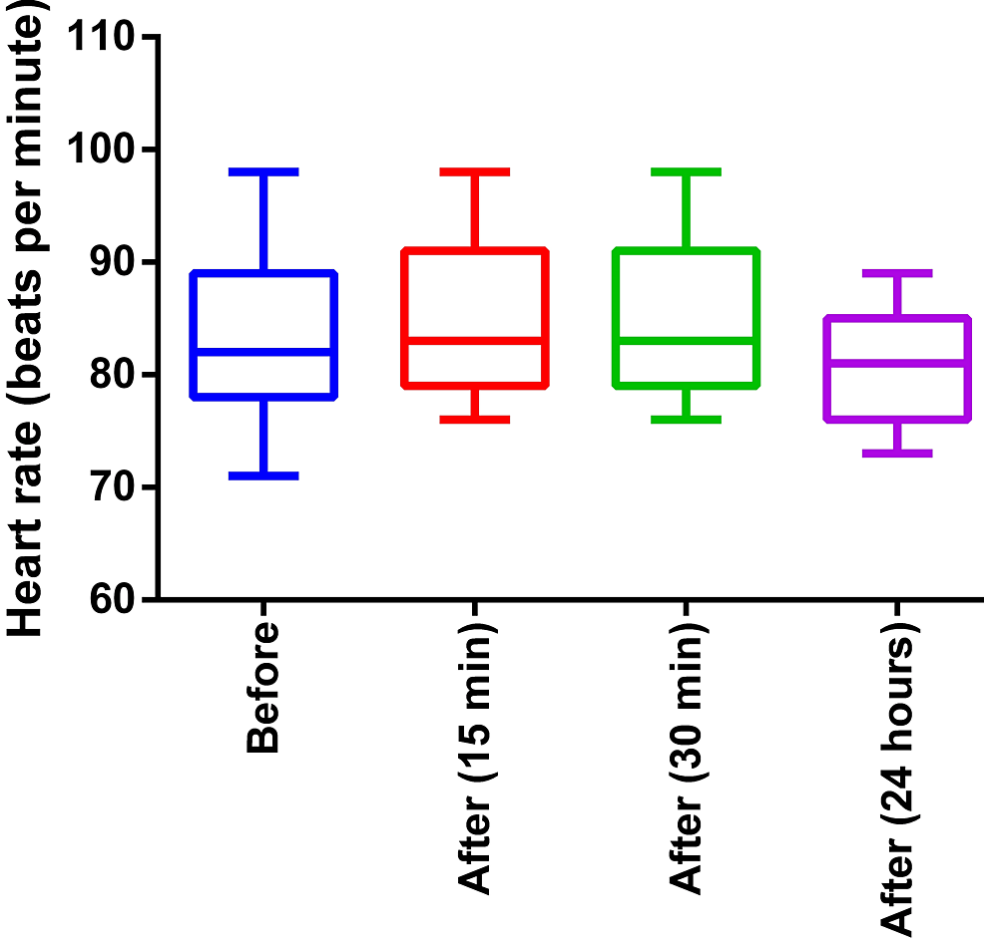
Heart rate before and after the intervention (three-month weight training program) Before: Before the exercise program, After: After the exercise program of three months (time in bracket indicates measurement after a session of exercise)

In the study group, the HR (p = 0.31) did not show any changes when measured before and after the intervention with 15 minutes, 30 minutes, and 24 hours rest (Figure 2).

**Figure 3:**
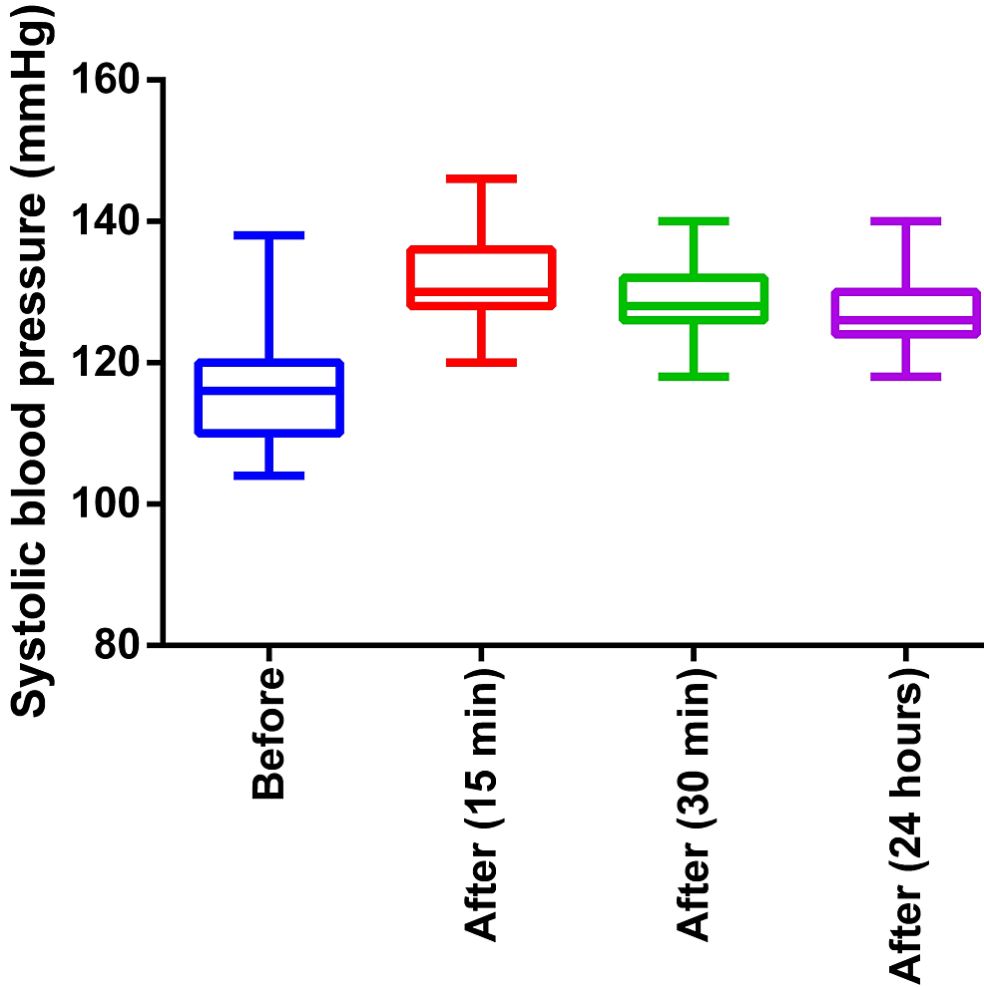
The systolic blood pressure before and after the intervention (three-month weight training program) Before: Before the exercise program, After: After the exercise program of three months (time in bracket indicates measurement after a session of exercise)

The systolic BP significantly increased after the intervention (median 116 mmHg before intervention versus 126 mmHg after intervention with 24 hours rest; Friedman p < 0.0001, post hoc pair statistically significant) as shown in Figure 3. In Dunn’s post hoc test, there was no significant difference between the systolic BP measured with 15 minutes rest and 30 minutes rest or between the systolic BP measured with 30 minutes rest and 24 hours rest.

**Figure 4:**
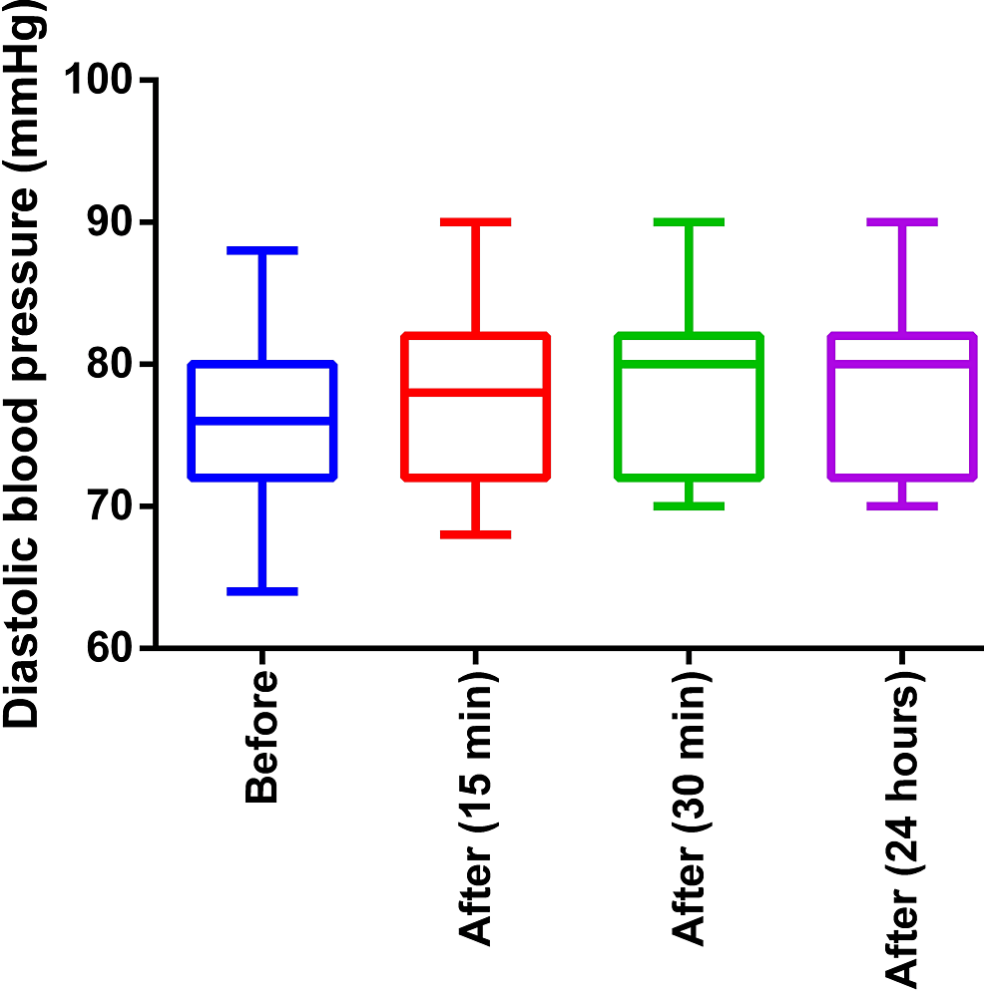
The diastolic blood pressure before and after the intervention (three-month weight training program) Before: Before the exercise program, After: After the exercise program of three months (time in bracket indicates measurement after a session of exercise)

Although in the Friedman test, the p-value was 0.025, Dunn’s multiple comparisons showed that there was no significant difference in any pair of data. Hence, the diastolic BP was not significantly changed after the intervention (median 76 versus 80 mmHg), as shown in Figure 4.

**Figure 5:**
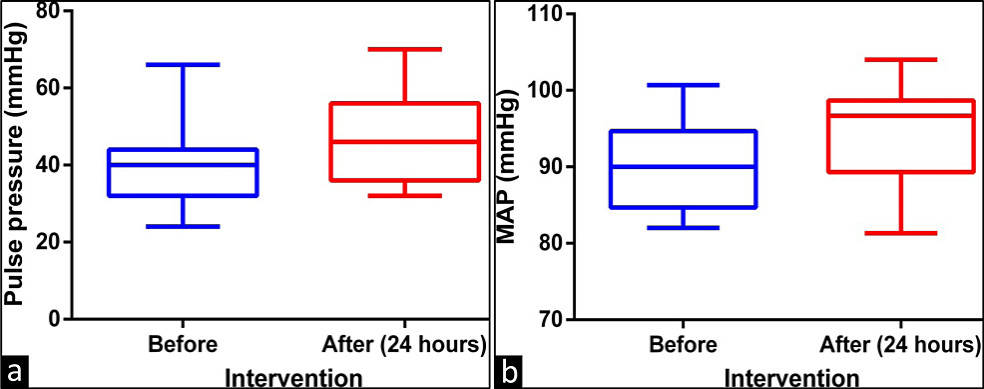
Comparison of pre-intervention and post-intervention (after 24 hours of rest) (a) pulse pressure and (b) mean arterial pressure MAP: Mean arterial pressure, Before: Before the exercise program, After: After the exercise program of three months (time in bracket indicates measurement after a session of exercise)

However, the pulse pressure (median 40 versus 46 mmHg, p = 0.025) and mean arterial blood pressure (median 90 versus 96.67 mmHg, p = 0.029) increased after the intervention in the study group, as shown in Figures 5a, 5b, respectively.

## Discussion

To find the effect of three-month weight training on the HR and BP among young adult males, we found that the HR and diastolic BP were not changed, but the systolic BP increased. The pulse pressure and mean arterial pressure were also increased. As a result, a muscle hypertrophy weight training program may raise systolic blood pressure in normotensive (including normal and elevated blood pressure according to the American Heart Association) young adult males [12]. In addition, the pulse pressure and mean arterial pressure were also increased. This indicates an overall increase in blood pressure during rest. A rest of 15 minutes may not be adequate for the cardiovascular system to come to a pre-exercise state; hence, we measured after 30 minutes of rest also. And finally, we measured the parameters after a 24-hour gap. This ensures that the immediate effect of exercise is not present during the measurement. In all three measurements, the systolic blood pressure was significantly higher.

There may be several reasons for the increase in blood pressure during exercise. The events occurring in weight training are an increase in muscle pressure, sympathetic vasoconstriction, and the accumulation of metabolites that increase sympathetic flow. The events cause an increase in peripheral resistance and increase cardiac output. This increases blood pressure during a weight training exercise [10]. However, these changes are transient due to the adaptation of the cardiovascular system to meet the extra demands of the body. After taking rest, the cardiovascular system is supposed to come to a pre-exercise state. In the long run, this lowers systolic blood pressure [13]. In our study, we found that a short-term exercise program does not cause a reduction in systolic blood pressure but increases it. And the increase is not only after exercise but is sustained after 24 hours. This discordant finding with other literature may be due to differences in sample age, physique, mode of exercise, duration of exercise, and variation in cardiometabolic changes [10,13,14]. However, the reason why this increment occurred should be a topic of future research that may include the extensive autonomic nervous system, metabolic changes, and vascular changes associated with weight training. However, we could not do it due to a lack of financial and resource limitations.

Physicians practicing medicine would encounter young adult male patients who may come with higher systolic blood pressure after weight training aimed at increasing hypertrophy of muscle. Hence, they should suggest the candidate combine weight training with other training modes for reducing weight training-induced high blood pressure [15].

Limitations

This study has several limitations. This study was a single-center study with a convenience sample of young adult males from a particular climate zone in India. Hence, the result may not be extrapolated to other young adults in India. The study was conducted only on males. Due to logistics (separate rooms, facilities, security) and manpower limitations, we could not include females. Hence, hemodynamic changes among males and females could not be compared. This study was done with a particular type of exercise program that is commonly practiced in the region for muscle hypertrophy. If other programs are applied, the result may not be corroborative. These should be considered in a future multi-centric study. In addition, although we asked the research participants to follow the same diet chart for both the study and control group, undeclared deviation, if any, may be a confounding factor. Furthermore, this study is not a randomized control trial which further limits the generalizability of the result.

## Conclusions

A short-term structured weight training program (that we used in this study) for three months period may sustain the increase in the systolic BP after a 15-minute, 30-minute, and 24-hour rest in young adult males. However, the diastolic BP remains similar to the pre-intervention level. The HR remains unchanged before and after the exercise program. Hence, lower sympathetic or higher parasympathetic control of the heart may not be found after a three-month weight training program. When young adults approach exercise trainers for weight training for muscle hypertrophy, they should be monitored frequently for changes in BP over time for any timely decision about the continuation of the program. Further studies are required to find the changes in the vasculature, muscle, and nerves that might have contributed to the higher systolic BP after the exercise program.
